# Dynamic changes of neutralizing antibody and memory T cell responses six months post Omicron XBB reinfection

**DOI:** 10.3389/fimmu.2024.1477721

**Published:** 2024-10-07

**Authors:** Xin-Jing Zhao, Xin-Lou Li, Sheng Zhang, Jin-Jin Chen, Wei-Chao Zhao, Na-Na Wu, Rui-Juan Wang, Qiang Xu, Chen-Long Lv, Bao-Gui Jiang, Guo-Lin Wang, Li-Qun Fang

**Affiliations:** ^1^ State Key Laboratory of Pathogen and Biosecurity, Academy of Military Medical Science, Beijing, China; ^2^ Department of Epidemiology and Biostatistics, School of Public Health, Anhui Medical University, Hefei, China; ^3^ Department of Medical Research, Key Laboratory of Environmental Sense Organ Stress and Health of the Ministry of Environmental Protection, the Ninth Medical Center, Chinese PLA General Hospital, Beijing, China; ^4^ Department of Respiratory Medicine, the Ninth Medical Center, Chinese PLA General Hospital, Beijing, China

**Keywords:** Omicron XBB reinfection, neutralizing antibody, memory T cell responses, immune persistence, cross-recognition

## Abstract

**Introduction:**

With the continued prevalence of COVID-19, repeated infection caused by SARS-CoV-2 has become common. However, studies on immune persistence post Omicron XBB reinfection are limited.

**Methods:**

We prospectively studied the durability and cross-reactivity of neutralizing antibodies (NAbs) and T cell responses among 20 subjects who suffered Omicron BA.5 infection with or without Omicron XBB reinfection over 6-month through the pseudovirus neutralization test and the fluorospot assay.

**Results:**

NAbs against EG.5.1, BA.2.86, and JN.1 subvariants were decreased and undetectable at 6-month post Omicron BA.5 infection, while those elicited by Omicron XBB reinfection were significant increased and remained detectable against all detected variants within 6-month. Furthermore, in subjects with Omicron XBB reinfection, memory T cell responses could cross-recognized wild-type and Omicron spike peptides and reached peak at 3-month. Interestingly, comparable robust T cell responses were observed among non-seroconverted subjects post Omicron XBB exposure.

**Conclusion:**

Though the NAbs against various emerging Omicron subvariants elicited by Omicron XBB reinfection can persist for at least 6-month, the HCWs should strengthen personal protection and timely be immunized with updated vaccines upon current circulating variants or conserved T epitope.

## Introduction

In China, with the termination of the zero-COVID-19 policy on December 07^th^ 2022, over 80% individuals were infected by Omicron BA.5 in the following two months (1). On May 05^th^ 2023, the World Health Organization declared the end of emergency phase of the Coronavirus Disease 2019 (COVID-19) pandemic (2). However, the continued evolution and transmission of severe acute respiratory syndrome coronavirus 2 (SARS-CoV-2) in the world has given rise to emerging new variants. Thus, between May and September 2023, a great many people contracted Omicron XBB subvariants in China (3).

Previous studies have demonstrated that the Omicron XBB sublineage (XBB.1, XBB.1.5, XBB.1.9, XBB.1.16, EG.5.1) and Omicron BA.2.86 sublineage (BA.2.86 and JN.1) extensively escape the immunity elicited by Wild-type (WT) vaccines, WT/BA.5 bivalent vaccines or previously non-XBB subvariants infection (4–6). Fortunately, Omicron XBB breakthrough infection (BTI) in human enhance the breadth and potency of cross-neutralization against Omicron XBB and BA.2.86 sublineages in the early convalescent (6–9). Similarly, the updated XBB.1.5 vaccines elicit potent neutralizing antibodies (NAbs) against the previous and contemporary SARS-CoV-2 variants, including Omicron XBB and BA.2.86 sublineages (10–12). However, the NAbs titers induced by Omicron XBB subvariants BTI are still relatively low against Omicron XBB and BA.2.86 sublineages, which may be influenced by the persisting immune imprinting raised by previous vaccination or infection (13, 14). Notably, the Omicron XBB subvariants reinfection may alleviate the WT-vaccination induced immune imprinting and can enhance the antibody responses approximately 1 month after the last infection (15).

Though immune resistance is observed against emerging SARS-CoV-2 variants in antibody responses, T cell responses show well cross-reactivity between different variants, including the pre-Omicron subvariants and Omicron subvariants (16), which play an important role in cleaning the virus and reducing the risk of hospitalization and/or severe illness (17, 18). Recent documented studies also demonstrate that virus-specific T cell responses against both WT and Omicron XBB variants are significantly enhanced in animal models and human after the immunization of recombinant spike protein or BNT162b2 mRNA XBB.1.5 vaccines (10, 11).

A previous study has found that healthcare workers (HCWs) and their households are at increased risk of contracting SARS-CoV-2 infection (19). Other studies also have shown that seroprevalence is significantly higher in HCWs than non-medical professions (20) and seronegative HCWs have stronger memory T cells compared to unexposed individuals (21). However, under the status of repeated epidemic waves of SARS-CoV-2, the characteristics of antibody and T cell responses post antigen exposure are worthy of further clarification.

Overall, previous studies have identified the humoral and cellular immunity after Omicron XBB vaccination or infection in the early stage, while our understanding of immunity persistence after Omicron XBB antigen exposure is limited. Therefore, we conducted a prospective study in a small cohort over 6-month following Omicron XBB reinfection to clarify the durability and cross-reactivity of NAbs and T cell responses.

## Materials and methods

### Study design and participants

**Figure 1:**
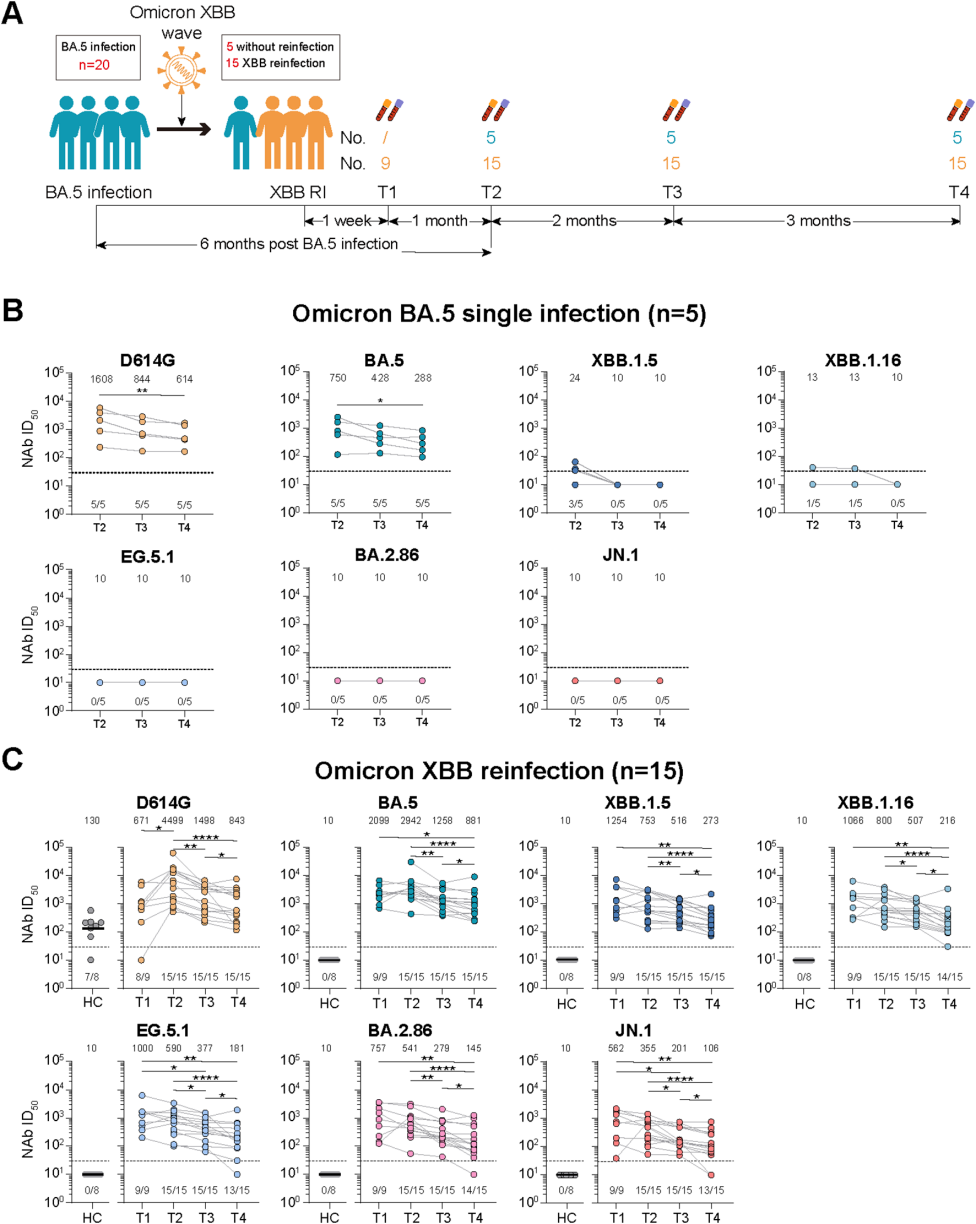
Study design and dynamic changes of neutralizing antibody responses against D614G and emerging Omicron subvariants among Omicron BA.5 infected participants with or without Omicron XBB reinfection. **(A)** Study design with enrolled participants, followed-up visited time points, blood sampling. **(B)** Dynamic changes of neutralizing antibody (NAb) titers against D614G and Omicron BA.5, XBB.1.5, XBB.1.16, EG.5.1, BA.2.86 and JN.1 at 6-, 8-, and 11-month post Omicron BA.5 reinfection among five participants. **(C)** Dynamic changes of NAb titers against D614G and Omicron BA.5, XBB.1.5, XBB.1.16, EG.5.1, BA.2.86 and JN.1 at 1-week, 1-, 3-, and 6-month post Omicron XBB reinfection among 15 participants. Eight vaccinated adults without prior SARS-CoV-2 infection were selected as healthy controls (HCs) in **(C)**. Values of geometric mean titer (GMT) were shown at the top of **(B, C)**. The positive no. and total no. were shown at the bottom of **(B, C)**. The black dashed line indicated the threshold for detectable NAb titers (ID_50_ = 30). Friedman test and Mann-Whitney test adjusted with FDR method were performed in **(B, C)** *P* values < 0.05 was considered statistically significant. *p<0.05, **p<0.01, ****p<0.0001.

Participants were enrolled from a hospital and an institute in Beijing between 22 May and 28 June when Omicron XBB subvariants were co-circulating in China (**Figure 1A**; **Supplementary Figure S1**). The characteristics of the participants (age, sex, occupation, vaccination background, infection status and clinical symptoms) are indicated in **Table 1**; **Supplementary Table S1**. Among the participants, there were nine HCWs (nurses and doctors) and eleven researchers (primary researchers and graduate students) enrolled in this study. Fifteen participants enrolled in the hospital and the institute showed COVID-19 symptoms within one month, while five subjects who worked together with the former in the same office of the institute but showed no COVID-19 symptoms were also enrolled at the same time. Investigation and sampling of the participants were conducted at 1-week, 1-month, 3-month, and 6-month, respectively. Each participant provided a 5-mL blood sample for serum isolation and an additional 10-mL blood sample for peripheral blood mononuclear cells (PBMCs) isolation at enrollment and follow-ups. Demographics at enrollment and previous COVID-19-like symptoms at follow-ups were collected from participants. Omicron BA.5 infection was confirmed by both epidemiological investigation and positive rRT-PCR or antigen test against SARS-CoV-2. Omicron XBB or EG.5.1 reinfection was confirmed by both epidemiological investigation and antigen test. Additionally, eight vaccinated subjects without previous infection by SARS-CoV-2 were enrolled as healthy controls (**Supplementary Table S2**).

**Table 1:** Characteristics of the participants.

Characteristics	Participants, n (%)
No.	20
Age (median, IQR)	32.0 (27.3-36.8)
Sex	
Male	7 (35.0)
Female	13 (65.0)
Occupation	
Researcher	11 (55.0)
Healthcare worker	9 (45.0)
Vaccination type	
Unvaccinated	1 (5.0)
1-dose BBIBP-CorV	1 (5.0)
3-dose ZF2001	1 (5.0)
2-dose Ad5-nCoV	6 (30.0)
3-dose CoronaVac/BBIBP-CorV	11 (55.0)
Infection status	
BA.5 single infection	5 (25.0)
BA.5 and XBB reinfection	15 (75.0)
Omicron BA.5 infection	
Yes	20 (100)
Symptomatic	20 (100)
Fever	20 (100)
Cough	18 (90.0)
Sore throat	17 (85.0)
Muscle aches	15 (75.0)
Runny nose	14 (70.0)
Headache	9 (45.0)
Taste or smell loss	9 (45.0)
Dizziness	8 (40.0)
Nasal congestion	7 (35.0)
Nausea	2 (10.0)
Omicron XBB infection	
Yes	15 (75.0)
Symptomatic	15 (100)
Fever	10 (66.7)
Sore throat	10 (66.7)
Runny nose	9 (60.0)
Muscle aches	8 (53.3)
Cough	7 (46.7)
Dizziness	4 (26.7)
Taste or smell loss	3 (20.0)
Nasal congestion	3 (20.0)
Headache	2 (13.3)
Nausea	2 (13.3)
Omicron EG.5.1 infection	
Yes	1 (5.0)
Symptomatic	1 (100)
Sore throat	1 (100)
Sampling time (median, IQR)	
Days between last vaccination and BA.5 infection^a^	358.0 (342.0-415.0)
Days between BA.5 infection and XBB reinfection^b^	157.0 (150.0-173.0)
Days between last infection (XBB/BA.5) and T1^c^	6.0 (4.0-10.0)/NA
Days between last infection (XBB/BA.5) and T2^c^	36.0 (31.0-39.0)/188.0 (181.5-199.0)
Days between last infection (XBB/BA.5) and T3^c^	96.0 (94.0-104.0)/253.0 (246.5-264.0)
Days between last infection (XBB/BA.5) and T4^c^	188.0 (177.0-193.0)/347.0 (335.5-353.0)

^a^For all participants (n=20); ^b^For participants who suffered Omicron XBB reinfection (n=15); ^c^For participants who suffered Omicron XBB reinfection (n=15) and Omicron BA.5 single infection (n=5), respectively. NA, not available.

### Serum and PBMCs isolation

Serum was isolated by centrifugation at 2500 rpm for 10 minutes, then aliquoted and preserved at -80°C until use. The PBMCs were isolated using SepMate^TM^-15 (STEMCELL Technologies Inc., Vancouver, Canada) density gradient centrifugation according to the manufacturer’s instructions. In Brief, blood samples, PBS+2% FBS, and density gradient medium were firstly mixed with equal volume. Then the diluted samples were centrifuged at 1200×g for 10 min at room temperature. PBMCs were poured into a separate 15 mL tube and washed twice with PBS+2% FBS. Finally, the PBMCs were resuspended with serum-free cell cryopreservation solution (New Cell & Molecular Biotech Co., Ltd, Cat# C40100) and stored at -80°C until use.

### Pseudovirus neutralization test

SARS-CoV-2 pseudoviruses were generated by co-transfecting HEK-293T cells (ATCC, Cat# CRL-3216) with human immunodeficiency virus backbones expressing firefly luciferase (pNL4-3-R-E-luciferase) and pcDNA3.1 vector encoding either D614G, BA.5, XBB.1.5, XBB.1.16, EG.5.1, BA.2.86, or JN.1 Spike proteins plasmid (**Supplementary Figures S2A**, **S3**) (9). Pseudoviruses were titrated on 293T-ACE2 cells (Vazyme, Cat# DD1701-01) prior to conducting the neutralization assays to normalize the viral input between assays. Heat-inactivated sera were serially diluted starting from 1:30 with a dilution factor of three. Then, 50 μL of diluted pseudovirus was added and incubated with diluted serum for 1 hour at 37°C. After that, 2x10^4^ 293T-ACE2 cells per well were added and incubated at 37°C, 5% CO_2_ for 48 hours. Subsequently, luciferase activity was quantified using the Luciferase Assay System (Vazyme, Cat# DD1204-02) using GloMax 96 Microplate Luminometer (Promega, E6521). Neutralization ID_50_ values for serum were calculated by a four-parameter nonlinear regression inhibitor curve in GraphPad Prism 8.0.2 (version 8.0.2, La Jolla, California, USA). A sample with ID_50_ values no more than 30 (the detectable limit) was considered negative for neutralizing antibodies and was assigned a value of 10 in geometric mean titer (GMT) calculations.

### Antigenic cartography

The neutralizing data of Omicron XBB reinfection was used to generate the antigenic map with R package Racmacs (version 1.1.9) and ggplot2 (version 3.4.2). The spacing of the grid lines corresponds to the neutralizing antibody titers unit, which was equivalent to the 2-fold change in the neutralizing ID_50_ titer.

### Fluorospot assays

To assess virus-specific memory T cells secreting IFN-γ or IL-2 against wild-type (WT) or Omicron BA.1 spike peptides, we performed Fluorospot assays using the SARS-CoV-2 (S+NMO) Human IFN-γ/IL-2 kit (FSP-0102-P12-1, Mabtech AB) and the SARS-CoV-2 (Omicron BA.1, S1 scan) Human IFN-γ/IL-2 kit (FSP-0102-P13-1, Mabtech AB) according to the manufacture’s protocols (**Supplementary Figure S2B**). In brief, the plates precoated with capturing mAbs (IFN-γ mAb 1-D1K and IL-2 mAb MT2A91/2C95) were washed with sterile PBS and blocked with RPMI 1640 culture media containing 10% fetal bovine serum (Gibico). 2.5x10^5^ PBMCs were added to each well and then stimulated by WT peptides (2 μg/mL), Omicron BA.1 peptides (2 μg/mL), or DMSO with anti-CD28 mAb (0.1 μg/mL) for 24 hours at 37°C in a 5% carbon dioxide atmosphere. Positive controls containing anti-CD3 mAb (0.1 μg/mL) and background controls were also included. After incubation, cells were discarded and the plates were washed with sterile PBS. Spots representing cytokine-secreting cells were detected by incubation with fluorophore-conjugated mAbs to IFN-γ or IL-2 followed by fluoroSpot enhancer, and spots were counted by the Mabtech IRIS FluoroSpot reader. To determine the virus-specific spots, the spots of the DMSO wells were subtracted from the WT or Omicron BA.1 peptides wells. The spots value above zero was considered as positive, and the spots value less than or equal to zero was determined as negative and assigned a value of 0.5 to facilitate drawing. Finally, the results were expressed as spot-forming cells (SFCs) per 10^6^ PBMCs.

### Statistical analysis

All statistical analyses were performed using GraphPad Prism (version 8.0.2) and RStudio (version 4.2.3). Differences between the groups were assessed by Mann-Whitney test, Friedman test or Wilcoxon test for matched data with FDR correction for multiple comparison. The strength of correlations was evaluated by Spearman’s test. And all statistical tests were 2-sided with a significance level of 0.05.

## Results

### Study participants and epidemiological characteristics

Twenty participants who were previously infected by Omicron BA.5 were enrolled in this study between May and July 2023 in Beijing when Omicron XBB subvariants were predominated in China (**Figure 1A**; **Supplementary Figure S1**). The median age of enrolled participants was 32.0 (interquartile range [IQR], 27.3-36.8), and seven (35.0%) were male. Eleven (55.0%) participants were researchers, and the other nine (45.0%) participants were HCWs. Of these participants, one (5.0%) individual was not vaccinated, while 19 (95.0%) participants partially or fully completed the primary or booster vaccination with several different COVID-19 vaccines. All participants suffered Omicron BA.5 infection, after which fever (100%), cough (90.0%), sore throat (85.0%), muscle aches (75.0%), and runny nose (70.0%) were the most reported symptoms. Fifteen participants (75.0%) suffered Omicron XBB infection, and fever (66.7%), sore throat (66.7%), runny nose (60.0%), and muscle aches (53.3%) were the most common symptoms. The median days between last vaccination and Omicron BA.5 infection of these participants were 358.0 (IQR, 342.0-415.0), and the median days between Omicron BA.5 infection and XBB reinfection were 157.0 (IQR, 150.0-173.0). What’s more, the median days between the last infection (XBB or BA.5 infection) and four different visited time points were 6.0 (IQR, 4.0-10.0)/NA, 36.0 (IQR, 31.0-39.0)/188.0 (IQR, 181.5-199.0), 96.0 (IQR, 94.0-104.0)/253.0 (IQR, 246.5-264.0), and 188.0 (IQR, 177.0-193.0)/347.0 (IQR, 335.5-353.0), respectively (**Table 1**).

### Dynamic changes of neutralizing antibodies against D614G and Omicron subvariants

We defined the visited time points of participants at enrollment, 1^st^ follow-up, 2^nd^ follow-up, 3^rd^ follow-up after Omicron XBB wave as T1, T2, T3, and T4, respectively (**Figure 1A**). We firstly measured the NAb titers of serum from participants against the D614G and various emerging Omicron subvariants (**Supplementary Figures S2A**, **S3**).

For participants with Omicron BA.5 single infection, we found that the NAb titers against D614G and Omicron BA.5, XBB.1.5, and XBB.1.16 dropped continuously from T2 to T4, while the NAb titers against Omicron EG.5.1, BA.2.86, and JN.1 subvariants were undetectable at all examined time points (**Figure 1B**). When compared the NAb titers against all detected SARS-CoV-2 variants at the same time point, we found that the NAb titers against Omicron XBB and BA.2.86 sublineages were obviously reduced than that against Omicron BA.5 (**Supplementary Figure S4A**).

For participants with Omicron XBB reinfection, when compared the NAbs titers among different examined points, we found that the NAb titers against the D614G and Omicron BA.5 were higher at T2 than T1, and then significantly reduced from T2 to T4, while the NAb titers against Omicron XBB sublineage (XBB.1.5, XBB.1.16 and EG.5.1) and BA.2.86 sublineage (BA.2.86 and JN.1) continuously decreased and remained detectable for majority of the participants from T1 to T4 (**Figure 1C**). Notably, for NAb titers against all Omicron subvariants, no significant difference was found between T1 and T2, while significant difference was found among T2, T3, and T4 (**Figure 1C**). We then compared the NAb titers against different SARS-CoV-2 variants at the same time points (**Supplementary Figure S4B**). At T1, the NAb titers against Omicron BA.5 were highest among all detected variants, while the NAb titers against D614G and other Omicron subvariants were decreased compared to Omicron BA.5, with reduction of 1.7-3.7 times. At T2, T3, and T4, comparable NAb titers were observed against both D614G and Omicron BA.5, while the NAb titers against Omicron XBB and BA.2.86 sublineages were significantly reduced compared to that against Omicron BA.5, among which the Omicron JN.1 exhibited more neutralization resistance than others. Further antigenic analysis showed that the Omicron BA.2.86 sublineage was distinctly different from the Omicron XBB sublineage, and the antigenic distances between D614G and Omicron subvariants continuously increased from T1 to T3, and then were shortened at T4 (**Supplementary Figure S4C**).

Additionally, considering the reinfection status, the participants with Omicron XBB reinfection had significant higher NAb titers against Omicron XBB and BA.2.86 sublineages compared to those with Omicron BA.5 single infection (**Supplementary Figure S5A**).

### Dynamic changes of virus spike-specific memory T cells against WT or Omicron peptides

**Figure 2:**
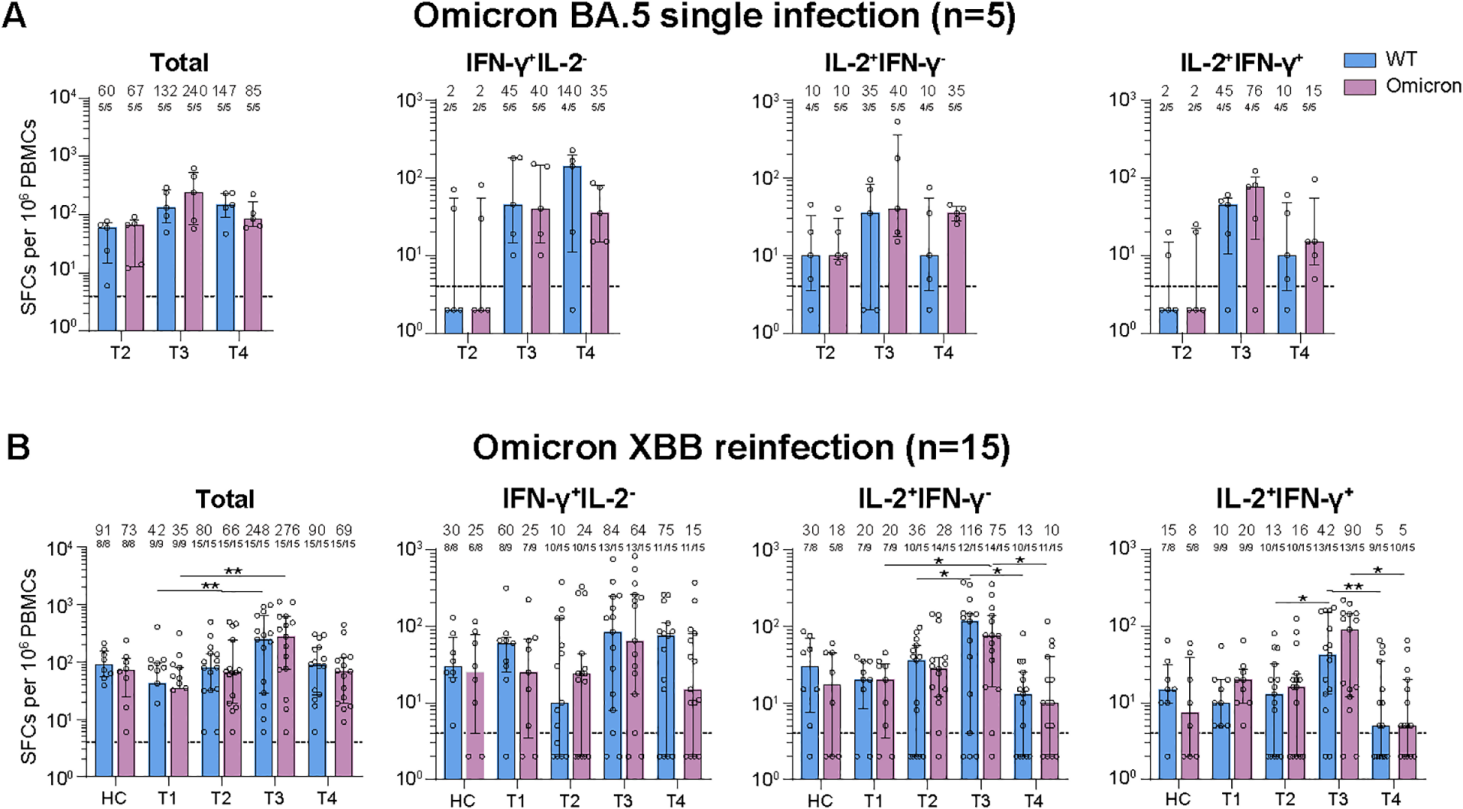
Dynamic changes of virus-specific memory T cell responses against wild-type (WT) and Omicron spike-derived peptides among Omicron BA.5 infected participants with or without Omicron XBB reinfection. **(A)** Dynamic changes of WT and Omicron spike-specific memory T cells (total, IFN-γ^+^IL-2^-^, IL-2^+^IFN-γ^-^, or IL-2^+^IFN-γ^+^) at 6-, 8-, and 11-month post Omicron BA.5 reinfection. **(B)** Dynamic changes of WT and Omicron spike-specific memory T cells (total, IFN-γ^+^IL-2^-^, IL-2^+^IFN-γ^-^, or IL-2^+^IFN-γ^+^) at 1-week, 1-, 3-, and 6-month post Omicron XBB reinfection among 15 participants. Values of median and proportion of SFCs (spot-forming cells) above 4 respectively were shown in the **(A, B)**. The black dashed line indicated the threshold for detectable T cell responses (SFCs/10^6^ PBMCs=4) in **(A, B)**. The bar indicate median and interquartile range (IQR). Wilcoxon matched-pairs signed rank test, Friedman test and Mann-Whitney test adjusted with FDR method were performed in **(A, B)** *P* values < 0.05 was considered statistically significant. *p<0.05, **p<0.01. HC, healthy control.

We then detected the spike-specific memory T cell responses against the WT or Omicron peptides (**Supplementary Figure S2B**). For participants with Omicron BA.5 single infection, though no significant difference were found for virus-specific cytokine secreting memory T cell responses among examined time points, the memory T cell responses were slightly higher at T3 than those at T2 and T4 except for the total or IFN-γ^+^IL-2^-^ T cell responses against the WT peptides (**Figure 2A**). What’s more, the total and cytokine specific (IFN-γ^+^IL-2^-^, IL-2^+^IFN-γ^-^, or IFN-γ^+^IL-2^+^) memory T cell responses were comparable against WT or Omicron peptides at all time points visited (**Figure 2A**).

For participants with Omicron XBB reinfection, the total and cytokine specific (IFN-γ^+^IL-2^-^, IL-2^+^IFN-γ^-^, or IL-2^+^IFN-γ^+^) memory T cell responses against both WT and Omicron peptides were comparable with that of healthy vaccinated controls at T1, T2, and T4, while the T cell responses were obviously higher at T3 than the other visited time points (**Figure 2B**). Though strength of T cell responses dynamic changed during the follow-up, most of the participants kept positive T cell responses (**Figure 2B**). Further comparison revealed that the memory T cell responses were similar and well-recognized against WT or Omicron peptides at each time points visited (**Figure 2B**).

Given the reinfection status, we further compared the memory T cell responses between participants with or without XBB reinfection (**Supplementary Figure S5B**). No significant difference of memory T cell responses were observed between the two groups at all examined time points, which was distinctly different from the change of NAb responses.

### Occupational exposure enhances the antibody responses and increases the susceptibility to reinfection

**Figure 3:**
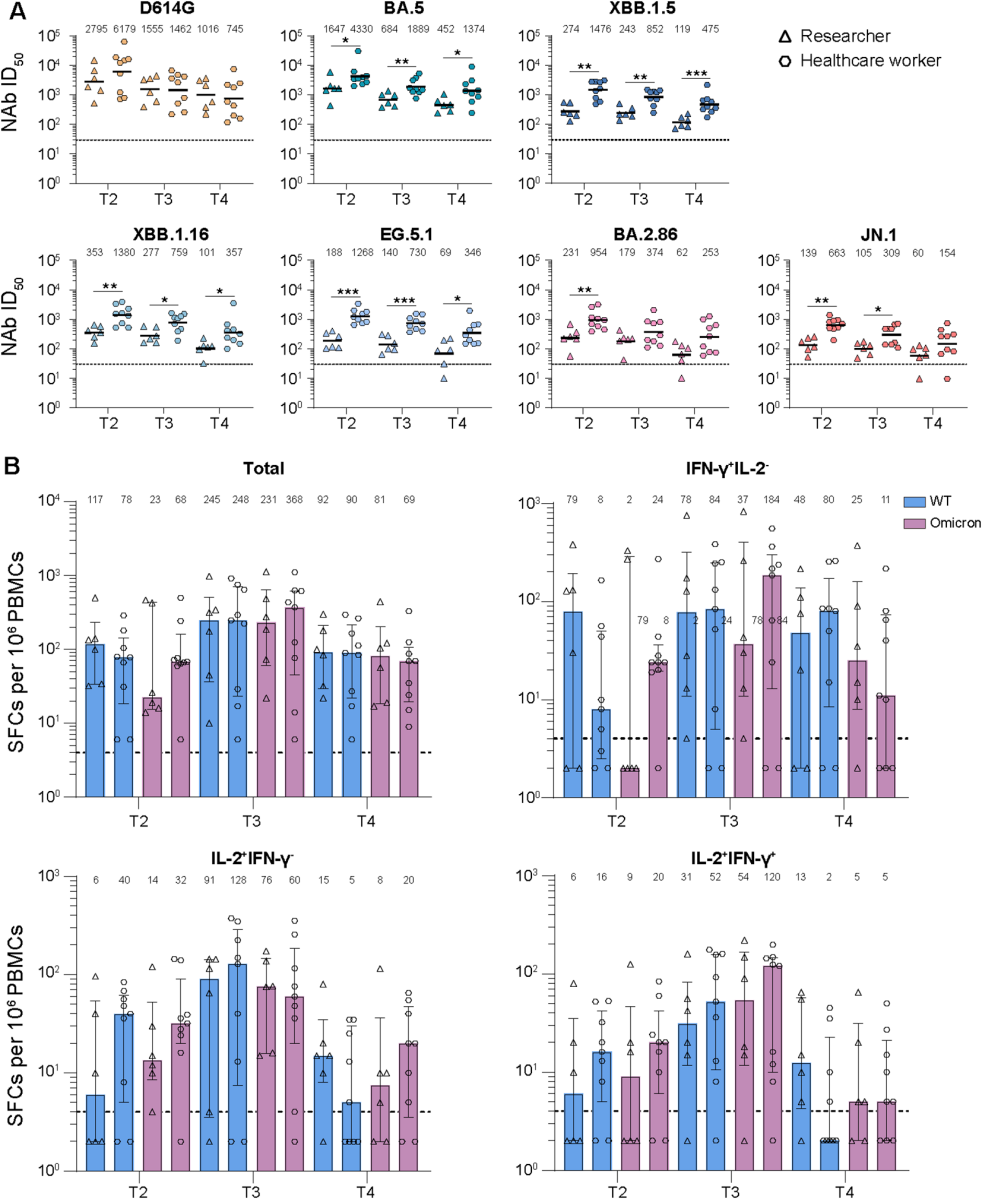
Comparison of neutralizing antibody and T cell responses between healthcare workers and researchers by different visited time points. **(A)** Comparison of neutralizing antibody (NAb) titers against various SARS-CoV-2 variants between healthcare workers and researchers. **(B)** Comparison of virus-specific memory T cell responses against wild-type (WT) or Omicron peptides between healthcare workers and researchers. Values of geometric mean titer (GMT) and median were shown at the above of **(A, B)**, respectively. The black dashed lines indicated the threshold for detectable NAb titers (ID_50_ = 30) in **(A)** and for positive T cell responses (SFCs/10^6^ PBMCs=4) in **(B).** The bar in **(A)** indicated GMT. The bar in **(B)** indicated median and interquartile range (IQR). Mann-Whitney test was performed for comparison in **(A, B)** *P* values < 0.05 was considered statistically significant. *p<0.05, **p<0.01, ***p<0.001.

We next analyzed the factors of occupation and sex that may influence the strength of immune responses. Regarding the occupation, we found that the healthcare workers had higher NAb titers against all detected variants than that of researchers at all examined points, among which most of the comparisons were significant (**Figure 3A**). However, no significant differences were observed between healthcare workers and researchers for virus-specific memory T cell responses against WT or Omicron peptides (**Figure 3B**). Considering the sex, there were no observed significant differences between males and females for both antibody and T cell responses (**Supplementary Figures S6A, B**).

Notably, the patient numbered by 15 (P15) showed higher NAb titers against all detected variants at T4 than that at T3, with increase by 1.0-2.1 times (**Supplementary Figure S7A**). Differently, the T cell responses of P15 were obviously reduced by 3.0-14.4 times at T4 compared to that at T3 (**Supplementary Figure S7B**). Combined with the epidemiological data that the P15 exhibited SARS-CoV-2 positive result by antigen self-test and had a sore throat on October 11^th^, 2023 between T3 and T4, we speculated that the P15 was very likely to be reinfected by Omicron EG.5.1 strain (**Supplementary Table S1**, **Supplementary Figure S7C**).

### Correlations among parameters of antibody and T cell responses

**Figure 4:**
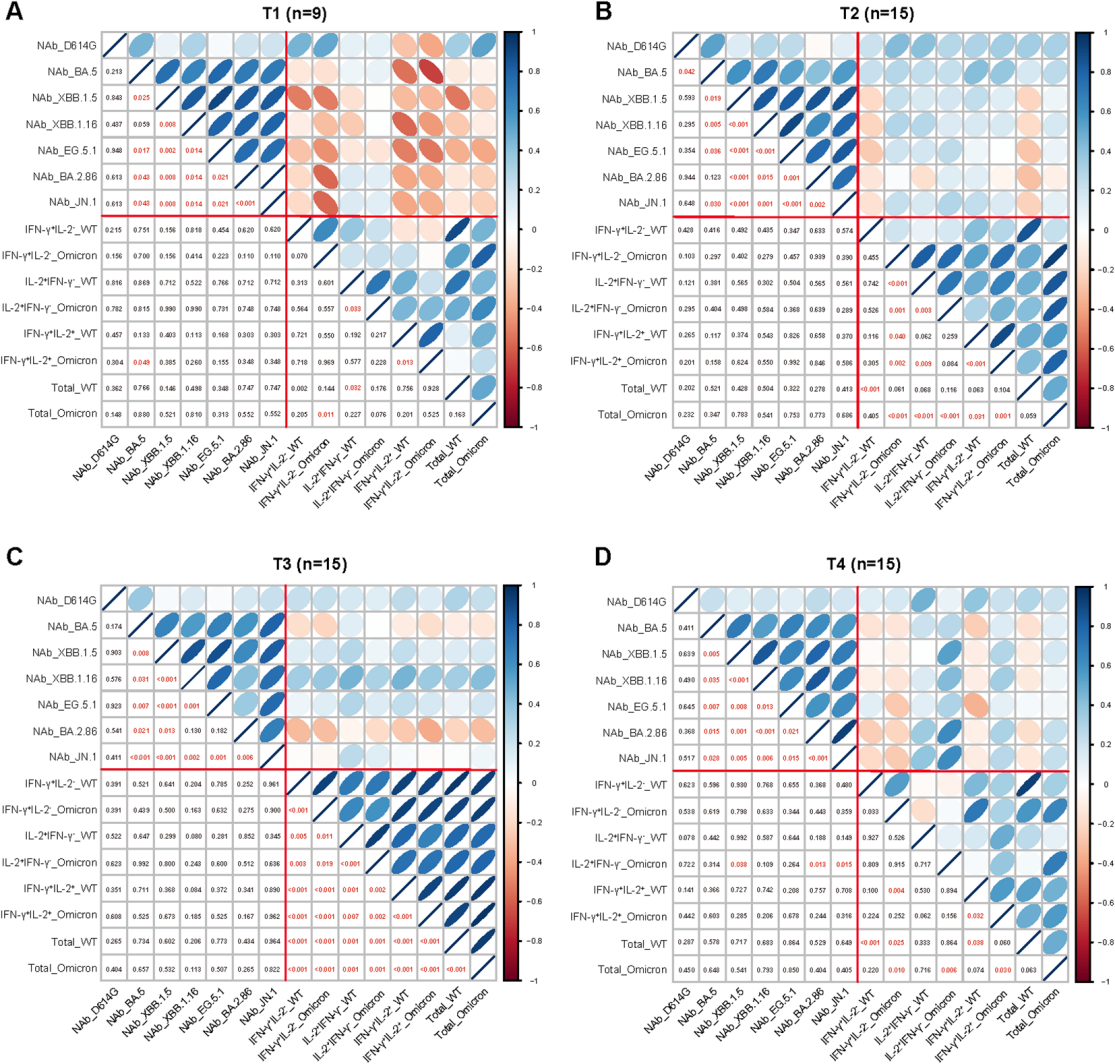
Correlation between the parameters of the adaptive immune response. Correlation between the parameters of antibody and T cell responses at 1-week **(A)**, 1-month **(B)**, 3-month **(C)**, and 6-month **(D)** post Omicron XBB reinfection. The strength of correlations was assessed by the two-side Sperman’s correlation test. Correlation strength was shown by the shape, color, orientation of the ellipse, and p value was shown by the number at the lower left. Red lines separated correlations between different measured immune parameters (antibody responses and T cell responses).

To explore whether correlations among detected immune parameters, we correlated the measured immune parameters including NAbs responses against various SARS-CoV-2 variants and virus spike-specific memory T cell responses at different visited time points (**Figures 4A–D**). There were significant positive correlations among the NAb titers of BA.5, XBB.1.5, XBB.1.16, EG.5.1, BA.2.86 and JN.1 from T1 to T4, while no significant correlations were observed between the NAb titers of the D614G and each of the Omicron subvariants. In addition, significant positive correlations among parameters of T cell responses were observed at T2 and T3. Surprisingly, we found weak negative correlations of parameters between antibody and T cell responses at T1.

## Discussion

In this study, we investigated the dynamic changes and cross-reactivity of humoral and cellular immunity after Omicron BA.5 infection with or without subsequent Omicron XBB reinfection among 20 participants during 6-month followed-up period. Our findings showed that the NAb titers against Omicron XBB and BA.2.86 sublineages were continuously reduced but most of them remained detectable during 6-month period post Omicron XBB reinfection. Differently, the T cell responses reached the peak at 3-month and rapidly decreased to the initial level at 6-month post Omicron XBB reinfection. Importantly, well cross-recognized memory T cell responses were found against WT and Omicron peptides. Additionally, Omicron XBB reinfection could enhance both the antibody and T cell responses, while Omicron XBB exposure without infection could only boost the T cell responses. Strong correlations were observed within parameters of antibody responses or T cell responses, respectively, while relatively weak negative correlations were found between NAb titers and T cell responses at 1-week post XBB reinfection. Healthcare workers had higher NAb titers compared to the researchers, however, they were still vulnerable to be attacked by the emerging Omicron subvariant.

Previous studies demonstrate that previous circulating SARS-CoV-2 variants BTI or Omicron XBB.1.5 vaccines immunization can elicit the NAb responses against the present circulating Omicron subvariants in the early stage (6–12, 22–24). A recent study in Denmark also reveals that the Omicron XBB.1.5 vaccine can provide a high level of protection in the short term, with a 76.1% reduced risk of COVID-19 hospitalization (25). However, due to immune imprinting (14), relatively lower NAb titers are observed against new emerging Omicron subvariants than the previous SARS-CoV-2 variants after Omicron XBB antigen exposure (6, 10). Importantly, combined with the previous study (15), we find that the Omicron XBB reinfection may alleviate the immune imprinting and enhance the antibody responses against Omicron XBB and BA.2.86 sublineages. Furthermore, we also identify that the NAb titers against Omicron XBB and BA.2.86 sublineages 1-month is lower than 1-week post Omicron XBB reinfection, while the NAb titers against D614G and Omicron BA.5 1-month is higher than 1-week post Omicron XBB reinfection, suggesting that the increasing speed of NAb responses against Omicron XBB and BA.2.86 sublineages is faster than that against D614G and Omicron BA.5 post Omicron XBB reinfection, and the growing speed of NAb responses may mostly depend on the last exposed SARS-CoV-2 variants. In addition, though the NAb titers reduced gradually, most of them remained detectable against the Omicron XBB and BA.2.86 sublineages 6-month post Omicron XBB reinfection, indicating that the Omicron XBB reinfection may provide relatively sufficient protection for the recovered participants against the infection by emerging Omicron subvariants. In fact, neutralizing antibodies are only one part of protective immunity against SARS-CoV-2, in addition to serum neutralization, the Fc-mediated effector functions of non-neutralizing antibodies are demonstrated significant importance to prevent severe COVID-19 (26, 27). However, limited by the conditions, no in-depth analysis for the non-neutralizing antibodies was made.

A previous study suggests that SARS-CoV-2 specific T cells are generated and remain present in convalescent patients 8 months after the primary infection (28). Another study demonstrates that a rapid and extensive recall of memory T cell populations occurs within one week after SARS-CoV-2 BTI (29). Differently, the virus-specific memory T cell responses are not enhanced within one month, obviously boosted at 3-month, and then decreased at 6-month post Omicron XBB reinfection in this study, revealing that the SARS-CoV-2 reinfection may delay the response speed and shorten the persistence of memory T cell response. Consistent with the previous study (16), well cross-recognized T cell responses are found against both WT- and Omicron-spike peptides post Omicron XBB reinfection in the study. Similarly, a recent study also finds that BNT162b2 XBB.1.5 vaccination can significantly enhance the T cell responses against both WT and XBB.1.5 peptides (10). Surprisingly, no significant difference of T cell responses is observed between subjects with Omicron XBB reinfection and subjects with Omicron BA.5 single infection in this study, which is clarified in a previous study that seronegative HCWs have stronger memory T cell responses after antigen exposure (21), suggesting that T cell responses may play an important role in preventing SARS-CoV-2 reinfection and Omicron XBB exposure could enhance T cell responses (30).

Previous studies report that HCWs have higher seroprevalence than the general population after the SARS-CoV-2 wave (31), and the occupation exposure is an important risk factor for SARS-CoV-2 infection (32). In this study, higher NAb titers are observed in HCWs than that in researchers, while the T cell responses were comparable between the two population groups, indicating that frequent occupational antigen exposure may enhance antibody responses rather than T cell responses. Furtherly, one HCW who have suffered Omicron XBB reinfection and possessed higher NAb titers against XBB subvariants are still reinfected by EG.5.1 in this study, suggesting that the HCWs are still vulnerable to be attacked by emerging Omicron subvariants.

There are several limitations of this study. First, a relatively small number of participants were enrolled. Second, we have not measured the non-neutralizing antibody, and focused more attention on spike-specific memory T cell responses rather than the other key proteins. Third, the fluorospot assays adopted are unable to furtherly distinguish virus-specific CD4^+^ or CD8^+^ T cells and PBMCs are stimulated with WT and Omicron BA.1 peptides, not including the XBB peptides. Therefore, more studies, including lager cohort of patients and various peptides against different variants for antibody and T cell responses, are needed to confirm and expand the results of this study in the future.

In conclusion, our study suggests that the NAb responses against the various emerging SARS-CoV-2 variants elicited by Omicron XBB reinfection can persist for at least 6-month among majority of the participants and memory T cell responses are able to cross-recognize both WT and Omicron peptides. Notably, the Omicron XBB exposure enhances both antibody and T cell responses, even among those non-seroconverted subjects. Furthermore, the HCWs have higher NAb responses than that of researchers, but they are still vulnerable to be attacked by the emerging Omicron subvariants. Therefore, updated vaccines upon the recent circulating variants or the conversed T cell epitope should be timely vaccinated among occupational population, especially the HCWs.

## Data Availability

The original contributions presented in the study are included in the article/**Supplementary Material**. Further inquiries can be directed to the corresponding authors.
