# Supplementary material for: Dynamic changes of neutralizing antibody and memory T cell responses six months post Omicron XBB reinfection
Source: Front Immunol. 2024 Oct 7;15:1477721. doi: 10.3389/fimmu.2024.1477721 (PMC11491401; doi:10.3389/fimmu.2024.1477721)
Supplement: Supplementary file 1 [file DataSheet1.docx]

**Supplementary Table S1**. **Characteristics of the COVID-19 patients enrolled in this study.**

| **Sample ID** | **Age** | **Sex** | **Occupation** | **Vaccine type** | **BA.5 infection** | |  | **XBB infection** | |  | **EG.5.1 infection** | |  | **Interval days** | | | | | |
| --- | --- | --- | --- | --- | --- | --- | --- | --- | --- | --- | --- | --- | --- | --- | --- | --- | --- | --- | --- |
|  |  |  |  |  | Yes/No | Date |  | Yes/No | Date |  | Yes/No | Date |  | Between last vaccination and BA.5 infection | Between BA.5 infection and XBB infection | Between BA.5/XBB infection and T1 | Between BA.5/XBB infection and T2 | Between BA.5/XBB infection and T3 | Between BA.5/XBB infection and T4 |
| 1 | 28 | Female | Researcher | Ad5-nCoV/Ad5-nCoV | Yes | 12/15/2022 |  | Yes | 05/08/2023 |  | No |  |  | 344 | 144 | 154/10 | 195/51 | 260/116 | 349/205 |
| 2 | 35 | Male | Researcher | Ad5-nCoV/Ad5-nCoV | Yes | 12/14/2022 |  | Yes | 05/19/2023 |  | No |  |  | 343 | 156 | 155/NA | 196/40 | 261/105 | 355/199 |
| 3 | 27 | Male | Researcher | CoronaVac/CoronaVac/CoronaVac | Yes | 12/13/2022 |  | Yes | 05/15/2023 |  | No |  |  | 397 | 153 | 156/NA | 197/44 | 262/109 | 351/198 |
| 4 | 24 | Female | Researcher | ZF2001/ZF2001/ZF2001 | Yes | 12/24/2022 |  | Yes | 05/23/2023 |  | No |  |  | 527 | 150 | 145/NA | 186/36 | 251/101 | 340/190 |
| 5 | 31 | Male | Researcher | Ad5-nCoV/Ad5-nCoV | Yes | 12/14/2022 |  | Yes | 05/20/2023 |  | No |  |  | 343 | 157 | 155/NA | 196/39 | 261/104 | 350/193 |
| 6 | 32 | Male | Researcher | Ad5-nCoV/Ad5-nCoV | Yes | 12/13/2022 |  | Yes | 05/21/2023 |  | No |  |  | 342 | 159 | 156/NA | 197/38 | 262/103 | 351/192 |
| 7 | 36 | Male | HCW | NA | Yes | 12/20/2022 |  | Yes | 05/19/2023 |  | No |  |  | NA | 150 | 153/3 | 182/32 | 241/91 | 335/185 |
| 8 | 35 | Female | HCW | BBIBP-CorV/BBIBP-CorV/BBIBP-CorV | Yes | 12/22/2022 |  | Yes | 05/16/2023 |  | No |  |  | 415 | 145 | 151/6 | 180/35 | 239/94 | 333/188 |
| 9 | 37 | Female | HCW | BBIBP-CorV/BBIBP-CorV/BBIBP-CorV | Yes | 12/20/2022 |  | Yes | 06/10/2023 |  | No |  |  | 413 | 172 | 182/10 | 203/31 | 267/95 | 349/177 |
| 10 | 40 | Female | HCW | BBIBP-CorV/BBIBP-CorV/BBIBP-CorV | Yes | 12/20/2022 |  | Yes | 06/11/2023 |  | No |  |  | 415 | 173 | 182/9 | 203/30 | 267/94 | 349/176 |
| 11 | 32 | Female | HCW | BBIBP-CorV/BBIBP-CorV/BBIBP-CorV | Yes | 12/23/2022 |  | Yes | 06/06/2023 |  | No |  |  | 416 | 165 | 179/14 | 200/35 | 264/99 | 346/181 |
| 12 | 38 | Female | HCW | BBIBP-CorV/BBIBP-CorV/BBIBP-CorV | Yes | 11/12/2022 |  | Yes | 06/10/2023 |  | No |  |  | 200 | 210 | 216/6 | 241/31 | 305/95 | 387/177 |
| 13 | 28 | Female | HCW | BBIBP-CorV/BBIBP-CorV/BBIBP-CorV | Yes | 11/12/2022 |  | Yes | 06/15/2023 |  | No |  |  | 192 | 215 | 216/1 | 241/26 | 305/90 | 387/172 |
| 14 | 41 | Female | HCW | BBIBP-CorV/BBIBP-CorV/BBIBP-CorV | Yes | 12/08/2022 |  | Yes | 07/12/2023 |  | No |  |  | 347 | 216 | 221/5 | 253/37 | 312/96 | 370/154 |
| 15 | 39 | Female | HCW | BBIBP-CorV/BBIBP-CorV/BBIBP-CorV | Yes | 12/21/2022 |  | Yes | 05/15/2023 |  | Yes | 10/11/2023 | | 398 | 145 | NA//NA | 181/36 | 233/88 | 337//192 |
| 16 | 29 | Female | Researcher | Ad5-nCoV/Ad5-nCoV | Yes | 12/13/2022 |  | No | NA |  | No |  |  | 342 | 158 | 156/NA | 197/NA | 262/NA | 351/NA |
| 17 | 23 | Male | Researcher | BBIBP-CorV/BBIBP-CorV/BBIBP-CorV | Yes | 12/29/2022 |  | No | NA |  | No |  |  | 425 | NA | 140//NA | 181//NA | 246//NA | 335//NA |
| 18 | 27 | Female | Researcher | BBIBP-CorV | Yes | 12/28/2022 |  | No | NA |  | No |  |  | 249 | NA | 141/NA | 182//NA | 247//NA | 336//NA |
| 19 | 33 | Male | Researcher | Ad5-nCoV/Ad5-nCoV | Yes | 12/09/2022 |  | No | NA |  | No |  |  | 395 | NA | 160/NA | 201//NA | 266//NA | 355//NA |
| 20 | 23 | Female | Researcher | CoronaVac/CoronaVac/BBIBP-CorV | Yes | 12/22/2022 |  | No | NA |  | No |  |  | 358 | NA | 147/NA | 188//NA | 253//NA | 347//NA |

HCW, healthcare worker. NA, not available.

**Supplementary Table S2**. **Characteristics of the vaccinated healthy controls enrolled in this study.**

| No. | Age | Sex | Vaccine type | Days between last vaccination and sampling |
| --- | --- | --- | --- | --- |
| 1 | 26 | Male | Ad5-nCoV/Ad5-nCoV | 401 |
| 2 | 23 | Male | BBIBP-CorV/BBIBP-CorV/BBIBP-CorV | 401 |
| 3 | 23 | Male | Ad5-nCoV/Ad5-nCoV | 401 |
| 4 | 24 | Male | BBIBP-CorV/BBIBP-CorV/BBIBP-CorV | 401 |
| 5 | 24 | Female | BBIBP-CorV/BBIBP-CorV/BBIBP-CorV | 363 |
| 6 | 25 | Female | BBIBP-CorV/BBIBP-CorV/BBIBP-CorV | 401 |
| 7 | 25 | Female | BBIBP-CorV/BBIBP-CorV/BBIBP-CorV | 401 |
| 8 | 27 | Female | Ad5-nCoV/Ad5-nCoV | 401 |


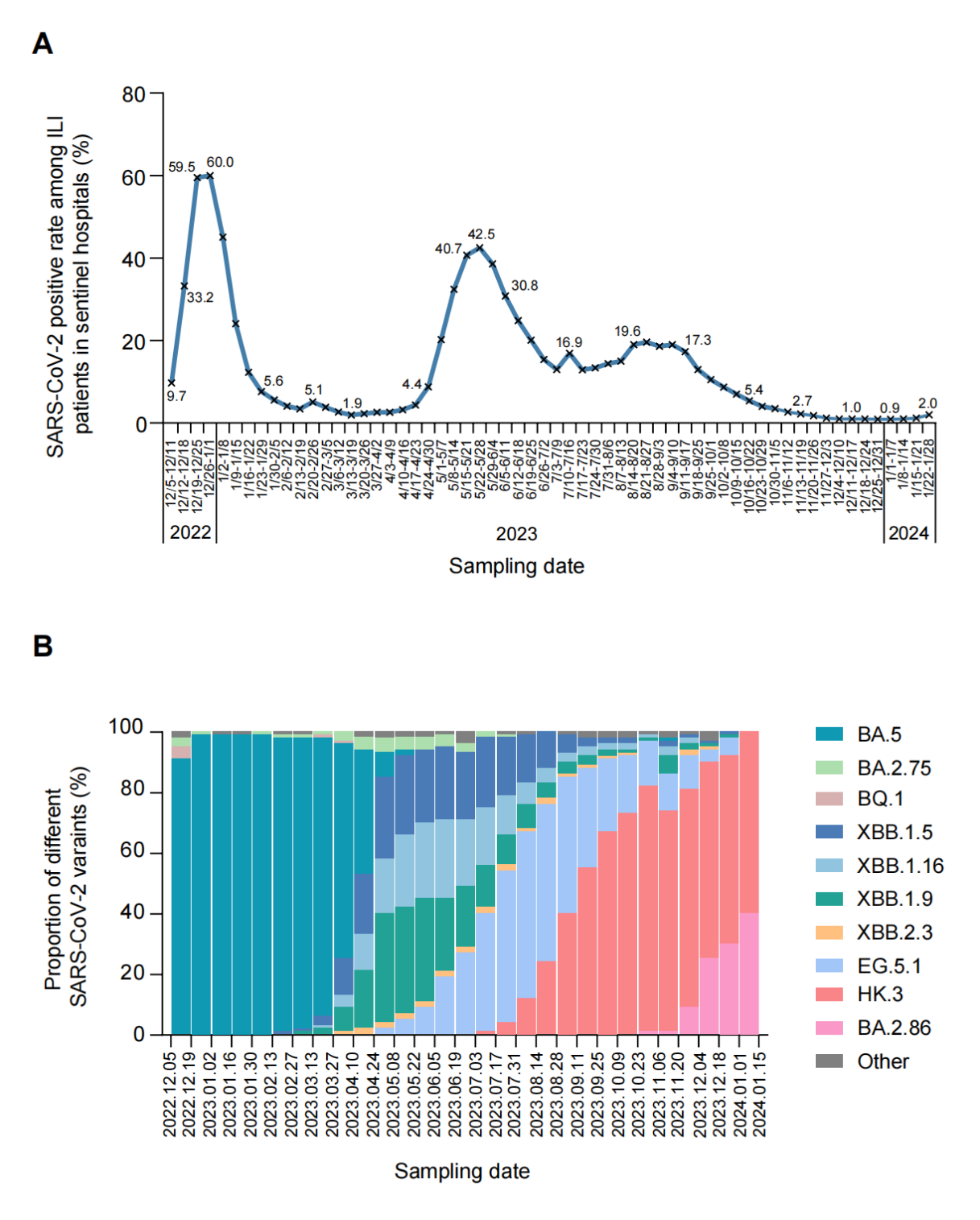


**Supplementary Figure S1. Surveillance data of COVID-19 in China.** (**A**) Surveillance data of SARS-CoV-2 positive rate among influenza-like illness (ILI) patients in sentinel hospitals between December 5th 2022 and January 28th 2024 in China. (**B**) Surveillance data of percentage of different SARS-CoV-2 variants between December 5th 2022 and January 15th 2024 in China. The surveillance data in panels A and B were downloaded from the database of China CDC (<https://www.chinacdc.cn/jkzt/crb/zl/szkb_11803/jszl_13141/index.html>) and GISAID (<https://covariants.org/per-country>), respectively. Related to Table 1 and Figure S1.


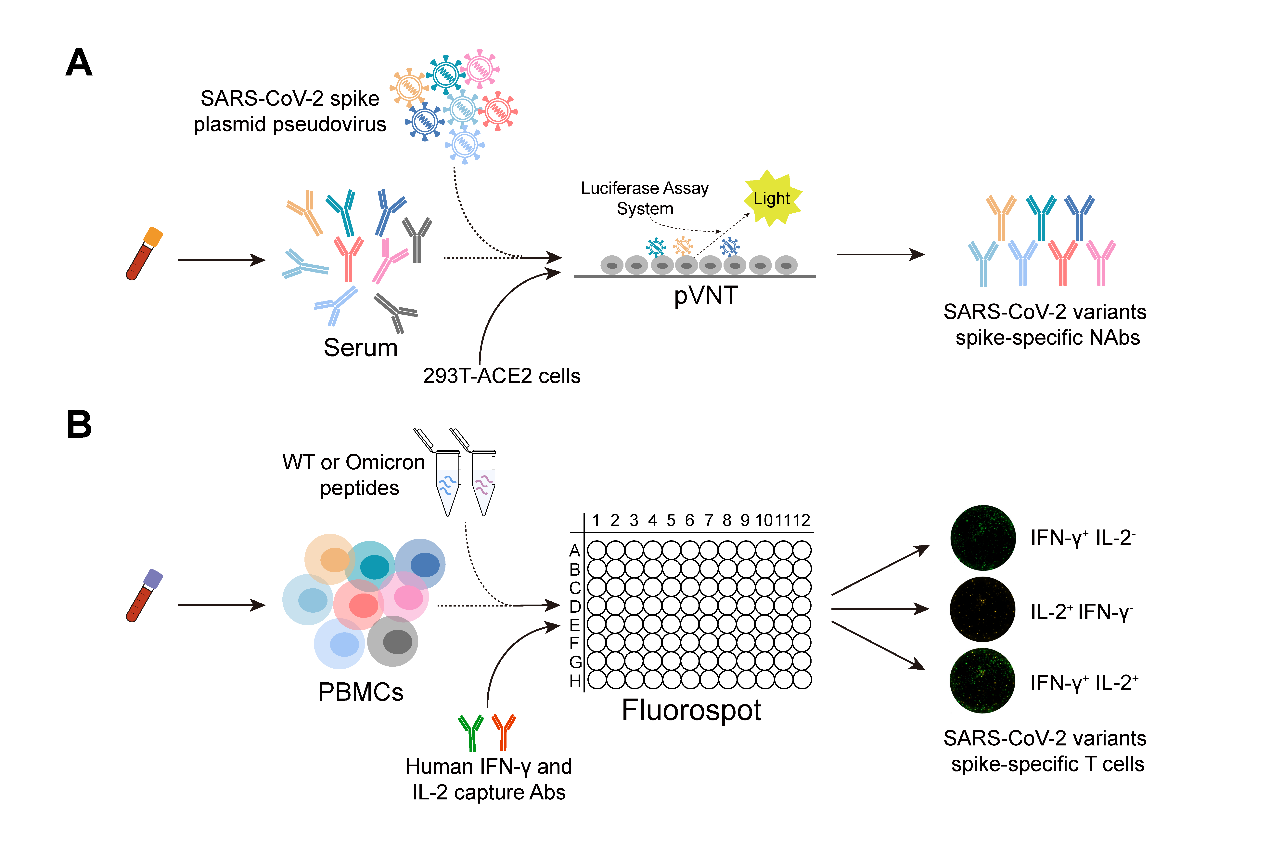


**Supplementary Figure S2. Study design and representative laboratory assays used in this study.** (**A**) Representative pseudovirus neutralization test (pVNT) used in this study. (**B**) Representative pseudovirus neutralization test (pVNT) and Fluorospot assays used in this study. Related to Figures 1 to 4


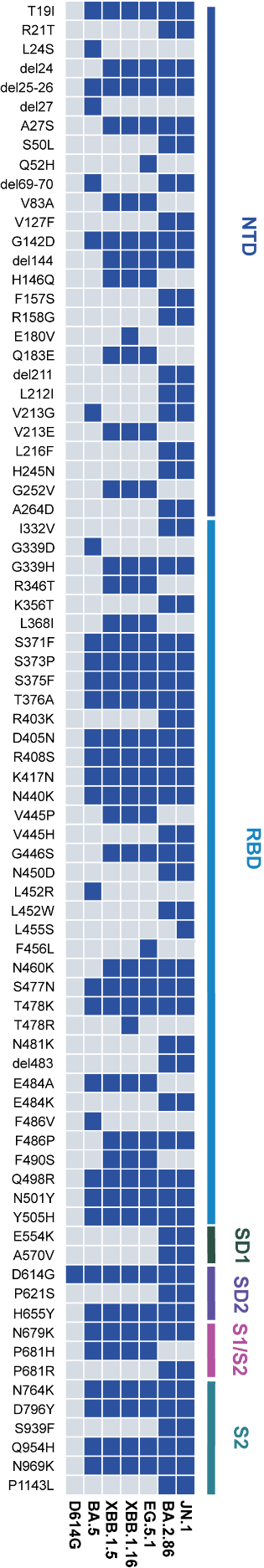


**Supplementary Figure S3. Mutations in spike plasmids of D614G and Omicron subvariants used in this study.**


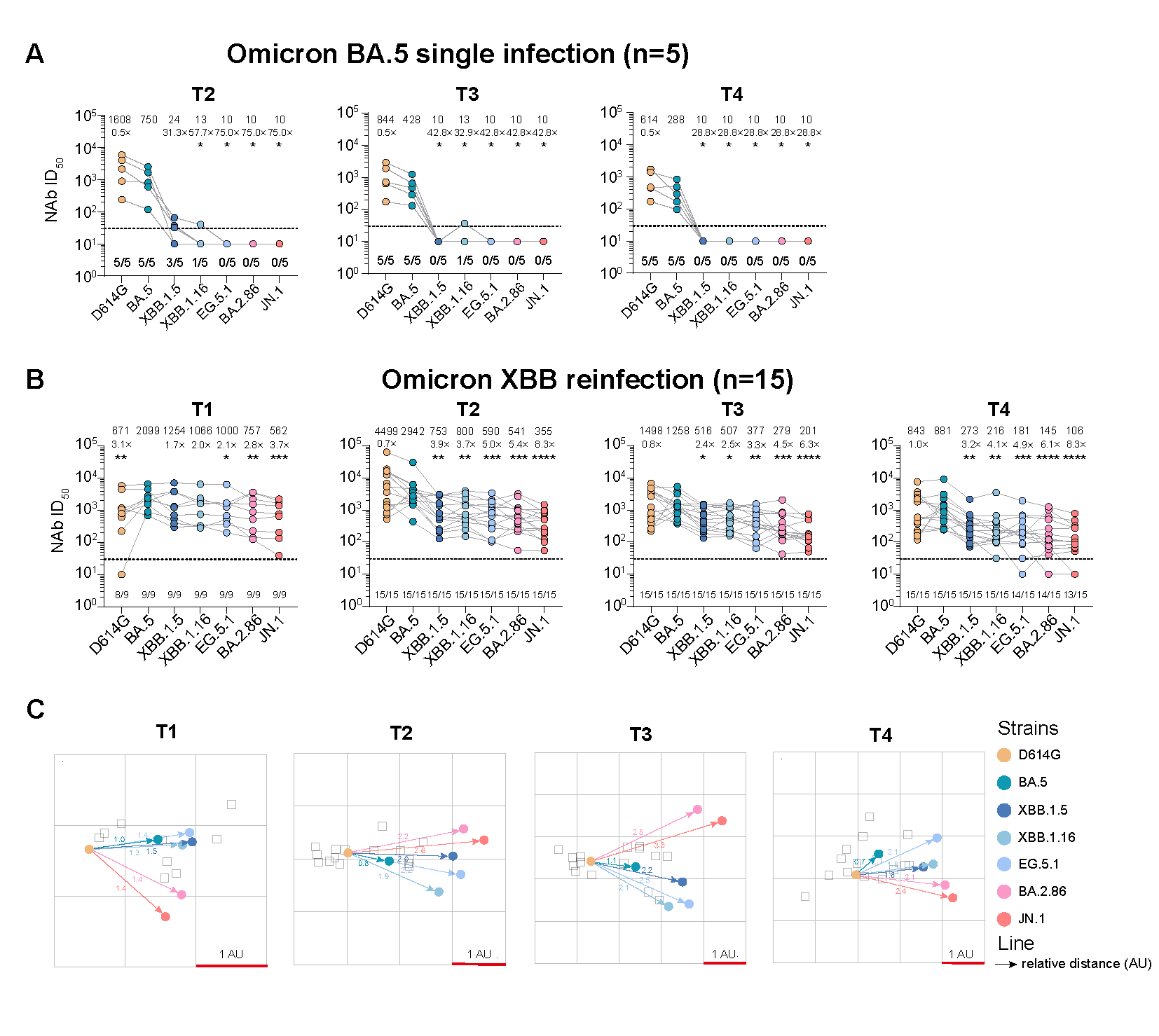
**Supplementary Figure S4. Neutralizing antibody responses against D614G and emerging Omicron subvariants**. (**A**) Comparison of NAb titers among five participants with BA.5 infectionagainst D614G and various emerging Omicron subvariants by different followed-up time points. (**B**) Comparison of NAb titers among 15 participants with XBB reinfection against D614G and various emerging Omicron subvariants by different followed-up time points. (**C**) Antigenic analysis with the NAb data among 15 participants with XBB reinfection at 1-week, 1-, 3-, and 6-month post Omicron XBB reinfection. Values of GMT with reduction times compared to BA.5 were shown at the top of panels A and B, and the proportion of detectable NAb titers above 30 were shown in the bottom of the panels A and B. The black dashed line indicates the threshold for detectable NAb titers (ID_50_=30). Friedman test adjusted with FDR method was performed in panels A B. *P* values<0.05 was considered statistically significant. **p*<0.05, ***p*<0.01, ****p*<0.001, **** *p*<0.0001.


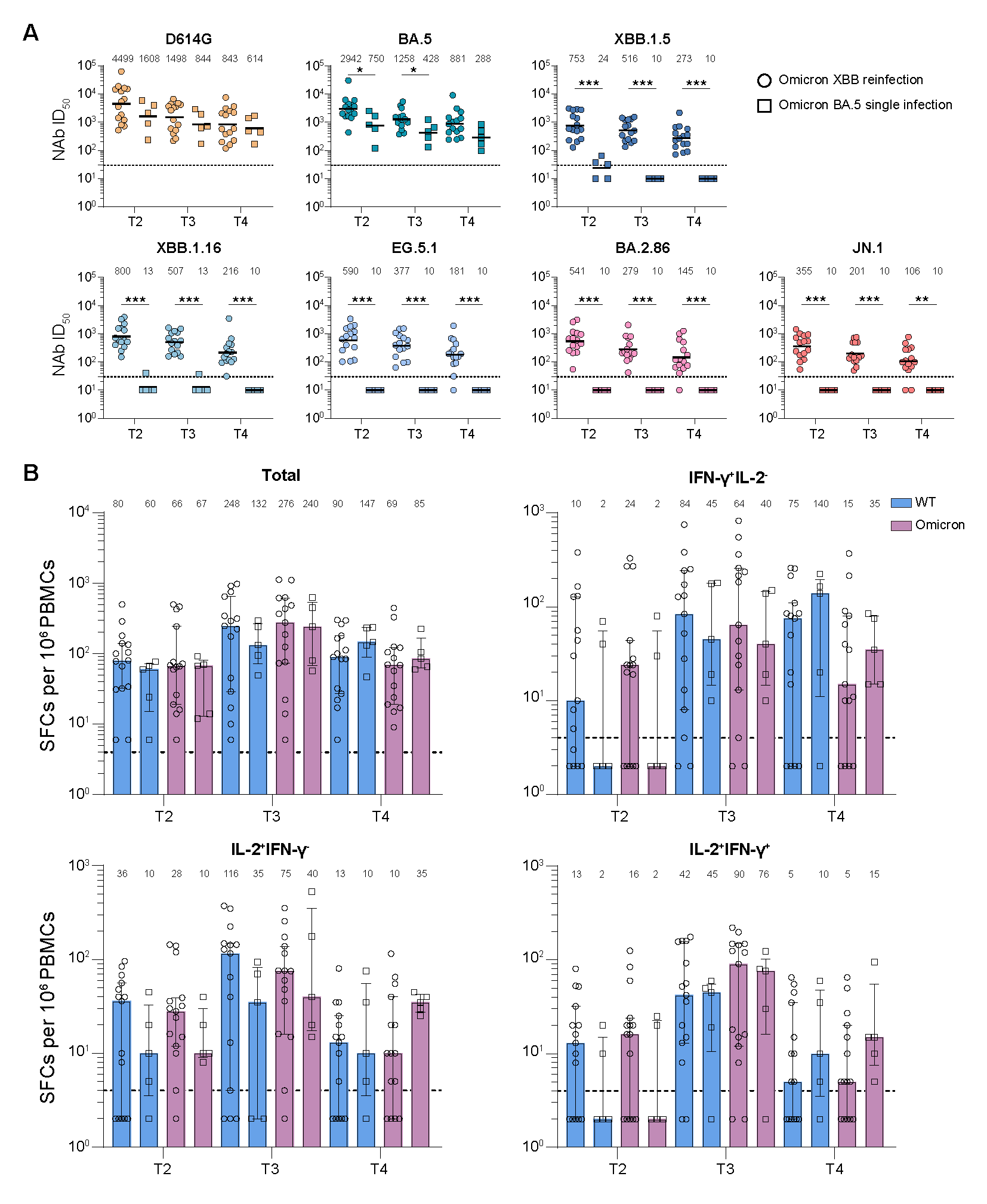


**Supplementary** **Figure S5.** **Comparison of neutralizing antibody and T cell responses between two groups by different reinfection status.** (**A**) Comparison of neutralizing antibody (NAb) titers against various SARS-CoV-2 variants between Omicron XBB reinfection group and Omicron BA.5 single infection group. (**B**) Comparison of virus-specific memory T cell responses against wild-type (WT) or Omicron peptides between Omicron XBB reinfection group and Omicron BA.5 single infection group. Values of geometric mean titer (GMT) and median were shown at the above of panel A and B, respectively. The black dashed lines indicated the threshold for detectable NAb titers (ID_50_=30) in panel A and for positive T cell responses (SFCs/10^6^ PBMCs=4) in panel B. The bar in panel B indicated median and interquartile range (IQR). Mann-Whitney test was performed for comparison in panels A and B. *P* values < 0.05 was considered statistically significant. **p*<0.05, ***p*<0.01, ****p*<0.001.


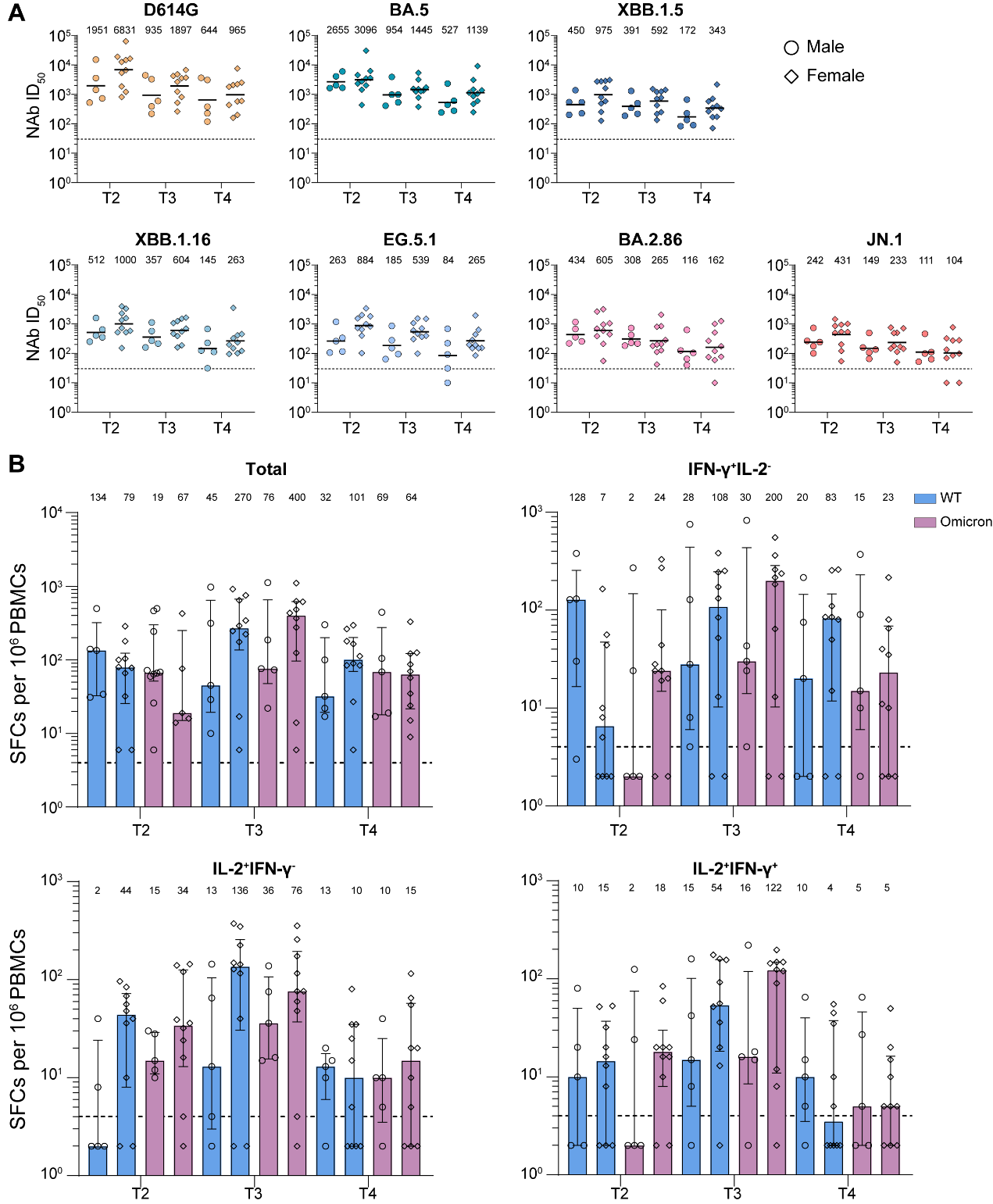


**Supplementary Figure S6.** **Comparison of neutralizing antibody and T cell responses between males and females by different visited time points.** (**A**) Comparison of neutralizing antibody (NAb) titers against various SARS-CoV-2 variants between males and females. (**B**) Comparison of virus-specific memory T cell responses against wild-type (WT) or Omicron peptides between males and females. Values of geometric mean titer (GMT) and median were shown at the above of panel A and B, respectively. The black dashed lines indicated the threshold for detectable NAb titers (ID_50_=30) in panel A and for positive T cell responses (SFCs/10^6^ PBMCs=4) in panel B. Boxplots indicated median and interquartile range (IQR). Mann-Whitney test was performed for comparison in panels A and B, and no significant difference with *p* values < 0.05 was found.


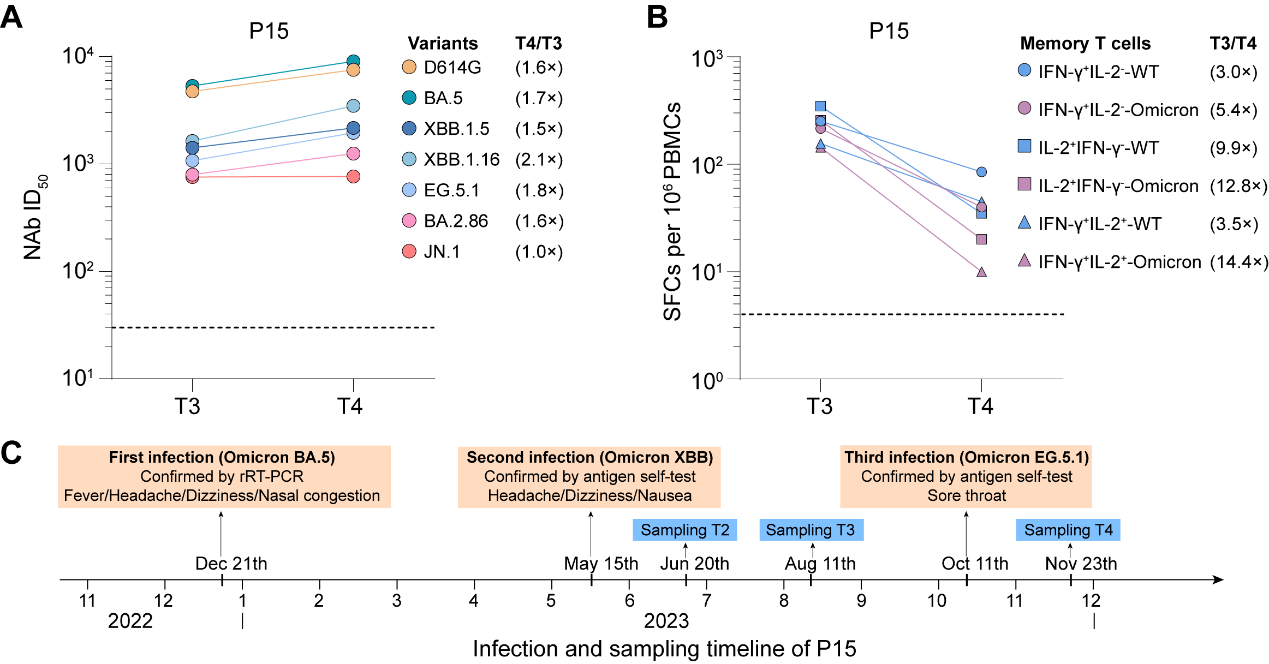


**Supplementary Figure S7. Immune response and epidemiological investigation of the participant with SARS-CoV-2 triple infection.** (**A**) Neutralizing antibody (NAb) responses against D614G and emerging Omicron subvariants of participant P15 at 3-month and 6-month post Omicron XBB reinfection. (**B**) Virus-specific memory T cell responses against wild-type (WT) or Omicron peptides of participant P15 at 3-month and 6-month post Omicron XBB reinfection. The black dashed lines indicated the threshold for detectable NAb titers (ID_50_=30) in panel A and for positive T cell responses (SFCs/10^6^ PBMCs=4) in panel B. Increase times of NAb titers (T4/T3) were shown at the right of panel A. Reduction times of memory T cell responses (T3/T4) were shown at the right of panel B. (**C**) Infection and sampling timeline of the participants P15 through epidemiological investigation.
